# Increased weighting on prior knowledge in Lewy body-associated visual hallucinations

**DOI:** 10.1093/braincomms/fcz007

**Published:** 2019-07-16

**Authors:** Angeliki Zarkali, Rick A Adams, Stamatios Psarras, Louise-Ann Leyland, Geraint Rees, Rimona S Weil

**Affiliations:** 1 Dementia Research Centre, University College London, 8-11 Queen Square, London WC1N 3AR, UK; 2 Max Planck Centre for Computational Psychiatry and Aging Research, University College London, 10-12 Russell Square, London WC1B 5EH, UK; 3 Department of Computer Science, University College London, Gower Street, London WC1E 6BT, UK; 4 Space Syntax Laboratory, University College London, 14 Upper Woburn Place, London WC1H 0NN, UK; 5 Institute of Cognitive Neuroscience, University College London, 17-19 Queen Square, London WC1N 3AR, UK; 6 Welcome Centre for Human Neuroimaging, University College London, 12 Queen Square, London WC1N 3AR, UK

**Keywords:** Visual hallucinations, Lewy body disease, predictive coding, visual perception, prior beliefs

## Abstract

Hallucinations are a common and distressing feature of many psychiatric and neurodegenerative conditions. In Lewy body disease, visual hallucinations are a defining feature, associated with worse outcomes; yet their mechanisms remain unclear and treatment options are limited. Here, we show that hallucinations in Lewy body disease are associated with altered integration of top-down predictions with incoming sensory evidence, specifically with an increased relative weighting of prior knowledge. We tested 37 individuals with Lewy body disease, 17 habitual hallucinators and 20 without hallucinations, and 20 age-matched healthy individuals. We employed an image-based learning paradigm to test whether people with Lewy body disease and visual hallucinations show higher dependence on prior knowledge. We used two-tone images that are difficult to disambiguate without any prior information but generate a strong percept when information is provided. We measured discrimination sensitivity before and after this information was provided. We observed that in people with Lewy body disease who experience hallucinations, there was greater improvement in discrimination sensitivity after information was provided, compared to non-hallucinators and controls. This suggests that people with Lewy body disease and hallucinations place higher relative weighting on prior knowledge than those who do not hallucinate. Importantly, increased severity of visual hallucinations was associated with an increased effect of prior knowledge. Together these findings suggest that visual hallucinations in Lewy body disease are linked to a shift towards top-down influences on perception and away from sensory evidence, perhaps due to an increase in sensory noise. This provides important mechanistic insights to how hallucinations develop in Lewy body disease, with potential for revealing new therapeutic targets.

## Teaser

The mechanisms of visual hallucinations in Lewy body disease are unknown. Zarkali *et al.* use two-tone stimuli to show that Lewy body hallucinations are associated with altered integration of top-down predictions with sensory evidence. Specifically, results show an increased relative weighting of prior knowledge in Lewy body hallucinations providing important mechanistic insights.

## Graphical Abstract

**Graphical Abstract:**
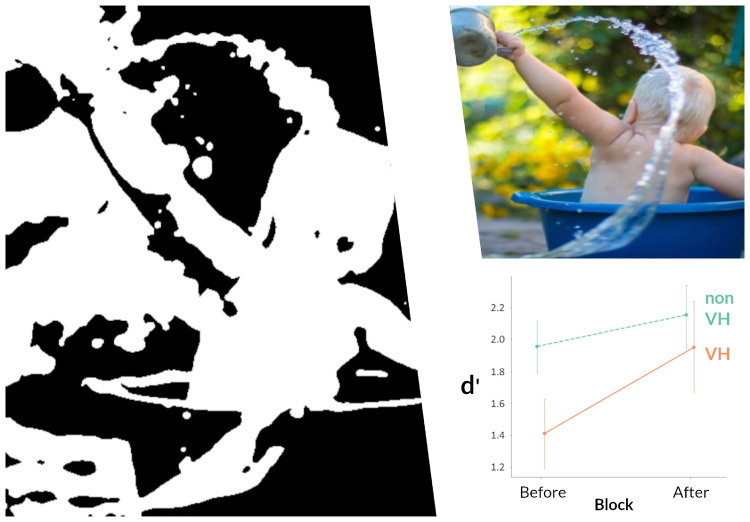

## Introduction

Hallucinations are commonly experienced in neurodegenerative diseases. They are particularly common and distressing in Lewy body disease (LBD: dementia with Lewy bodies and Parkinson’s disease), where they occur in up to 70% of patients and are associated with poorer outcomes (Fénelon *et al.*, 2000; Aarsland *et al.*, 2007; Weil *et al.*, 2016). Their phenomenology and severity vary, from illusions and misperceptions in the early stages, to complex, detailed and even animated imagery as the disease progress (Fénelon *et al.*, 2000; Gallagher *et al.*, 2011). Insight into whether experienced images are real or not is initially preserved but often withers during the course of the disease (Fénelon *et al.*, 2000; Weil *et al.*, 2016). Despite their high prevalence and impact on patients and carers, the mechanisms of hallucinations in LBD remain unclear and treatment options limited (Fénelon *et al.*, 2000; Weil *et al.*, 2016).

An attractive framework to understand hallucinations is predictive coding; here perception is seen as approximating Bayesian inference, a process with two key ingredients: expectations from prior knowledge and sensory input (Cavanagh, 2011, O’Callaghan *et al.*, 2017*b*; Parr *et al.*, 2018). In this context, visual hallucinations (VH), or experiencing visual percepts that are not objectively there, can be thought of as false inference which arises when the integration of sensory input and prior knowledge is altered (Fletcher and Frith, 2009; Adams *et al.*, 2013; Powers *et al.*, 2016; Corlett *et al.*, 2019). This framework has been applied to the study of hallucinations in non-neurodegenerative diseases. Hallucinations experienced by healthy individuals or in the context of psychiatric illness are associated with a shift towards prior knowledge and away from sensory evidence (Teufel *et al.*, 2015; Alderson-Day *et al.*, 2017; Powers *et al.*, 2017; Davies *et al.*, 2018). This relative increase in the weighting of prior knowledge in people who experience hallucinations has been linked with improved performance in disambiguating ambiguous visual and auditory signals (Teufel *et al.*, 2015; Alderson-Day *et al.*, 2017; Davies *et al.*, 2018) as well as a propensity to hallucinate when stimuli are noisy (Powers *et al.*, 2017).

Abnormal integration of priors with sensory information could also underlie hallucinations in neurodegeneration. In LBD, hallucinations are associated with visual-processing deficits (Diederich *et al.*, 2005; Weil *et al.*, 2016) and sensory evidence accumulation has been shown to be impaired in patients with Parkinson’s disease and VH (O’Callaghan *et al.*, 2017*a*). However, hallucinations can also occur in patients without any lower or higher visual deficits (Gallagher *et al.*, 2011; Weil *et al.*, 2016) making a solely bottom-up explanation for the occurrence of hallucinations less attractive.

Here, we tested the hypothesis that hallucinations in LBD are associated with an increased weighting of prior knowledge relative to sensory evidence. Given that hallucinations in LBD are almost always visual (Fénelon *et al.*, 2000; Aarsland *et al.*, 2007), we used a visual learning paradigm to test our hypothesis. We tested patients with LBD with and without hallucinations and unaffected age-matched controls on a paradigm that uses ambiguous sensory stimuli viewed before and after additional knowledge was provided. This paradigm allowed us to keep the sensory evidence constant but manipulate prior knowledge by supplying participants with information and comparing performance before and after information was provided. We hypothesized (i) that patients with LBD and VH would show greater improvement in performance compared to patients with LBD who did not experience hallucinations; (ii) that this performance benefit would be higher in those patients with more severe hallucinations.

In summary, we explored how prior knowledge is used during visual processing in people with LBD and VH. In accordance with our hypotheses, we have shown, for the first time, that hallucinations in neurodegeneration are associated with an increased relative weighting of prior knowledge over incoming sensory evidence. Importantly, we show that the degree of this prior weighting is proportionate to the severity of hallucinations. These findings provide important insights into the mechanisms of hallucinations in LBD and wider neurodegeneration and could inform future research into therapeutic targets.

## Materials and methods

### Experimental design and procedure

**Figure 1:**
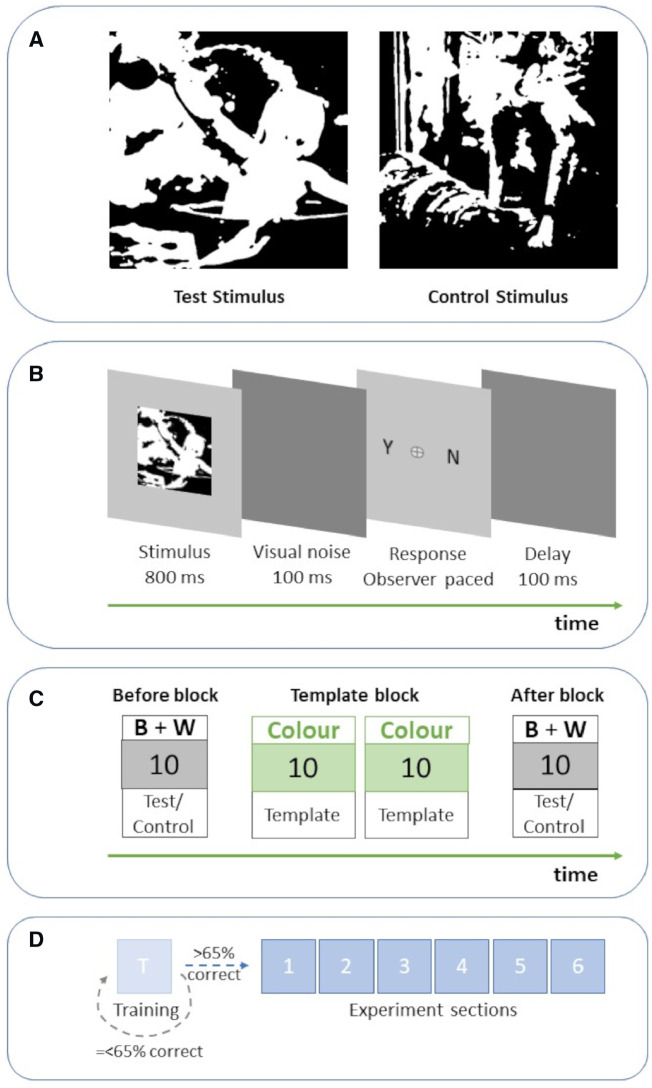
**Illustration of the experimental procedure.** (**A**) Example of a test stimulus (Left) and control stimulus (Right). Both have similar characteristics but the test images contain a person. (**B**) Individual trial: participants are briefly presented with an image and then are asked to indicate whether the image contains a person or not. Response was observer-paced without time limit to ensure capture from our clinical groups. (**C**) Experiment section: first, in the Before Block, participants are presented with 10 two-tone images (5 test stimulus and 5 controls), back-to-back in random order. Then, participants are presented with a Template block of 20 colour images in random order. All templates for the two-tone stimuli shown in Before Block are included. After each presented template image participants are asked again to indicate the presence of a person. This facilitates participant compliance via task simplicity and also ensures that participants actively observe the template images to provide participants with prior knowledge of the two-tone image content. Finally, in the After Block participants are presented with the same two-tone images as in Before Block. (**D**) Experiment: the experiment consists of six sections and starts with a training section, identical to the experimental sections but with two-tone images that are easier to disambiguate. Only participants with >65% discrimination sensitivity proceed to the main experiment.

**Figure 2:**
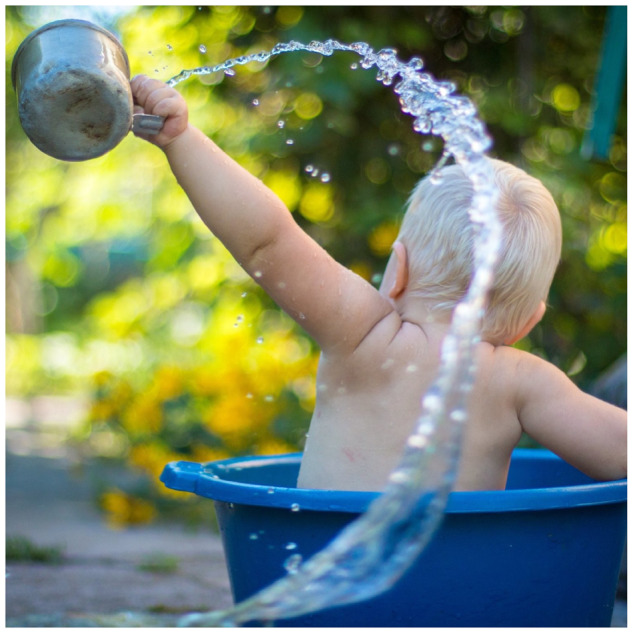
**Example of a template image.** This image was used to create the test stimulus in Figure 1A.

We used a visual learning paradigm with two-tone images as stimuli. When these are first seen without any prior knowledge, they appear as meaningless black and white patches (Fig. 1A) but when the template from which they were created is seen (Fig. 2) they generate a strong, coherent percept (Dolan *et al.*, 1997; Moore and Cavanagh, 1998; Hegdé and Kersten, 2010; Hsieh *et al.*, 2010; Cavanagh, 2011; Teufel *et al.*, 2015). The ability to disambiguate two-tone images with the aid of prior knowledge is mediated by top-down influences from high-level processes onto low-level visual function. This is supported by both psychophysics and neuroimaging studies (Dolan *et al.*, 1997; Hsieh *et al.*, 2010; Teufel *et al.*, 2015; Davies *et al.*, 2018).

To quantify the effect of prior knowledge in the different study groups, we compared how effectively the participants disambiguated the two-tone images before and after prior knowledge was supplied. We used a block design to supply participants with prior knowledge (Fig. 1C). First, in the ‘Before block’ participants were presented with 10 two-tone images, then in the ‘Template block’ participants were shown 20 coloured images (including the templates for the 10 two-tone images of the Before block to provide participants with prior knowledge of the two-tone image content) and finally in the ‘After block’ the 10 two-tone images of the Before block were repeated again. The order of specific images was randomized within each block. On each trial participants were presented with an image (two-tone or colour) for 800 ms. After each image presentation, we asked participants to indicate whether they could see a person in the image or not by keyboard press (Fig. 1B). Due to the expected prolonged reaction times of our clinical population the response screen was participant guided with no response time limit. We employed the same process during Template blocks, to ensure that participants were actively observing the template images thus receiving optimal prior information in preparation for the After block. In addition, keeping the same design across the whole experiment and including a training session at the onset ensured that our task was easy to understand and execute. This was of particular significance given the additional frailty and cognitive impairment in our cohort.

The experiment consisted of 6 sessions, each with the three blocks of trials described above, amounting to a total of 60 trials per participant all conducted on one study visit. The same set of 60 stimuli and 120 template images was used for each participant with order of presentation randomized in each block. The experiment started with a practice session identical to the experimental sessions; only participants achieving ≥65% discrimination sensitivity in the practice session proceeded to the experiment. This threshold was chosen to ensure that included participants were familiar with the procedure and understood the task. All of the invited participants in our study reached this threshold and were included in this analysis. After each experimental session participants were able to take a short break of maximum 5 min prior to continuing the experiment.

The experiment was conducted using a Dell XPS9570 15’ laptop with a 4k display at maximum brightness; stimuli were presented with a custom application made on Unity (version 2018.3.0b4) with participants seated at distance ∼60 cm from the screen. The experimental procedure is described in Fig. 1.

### Stimuli

We used two-tone images that are difficult to disambiguate without any prior information but once the template from which they were created is seen, they give the strong experience of a coherent percept.

**Figure 3:**
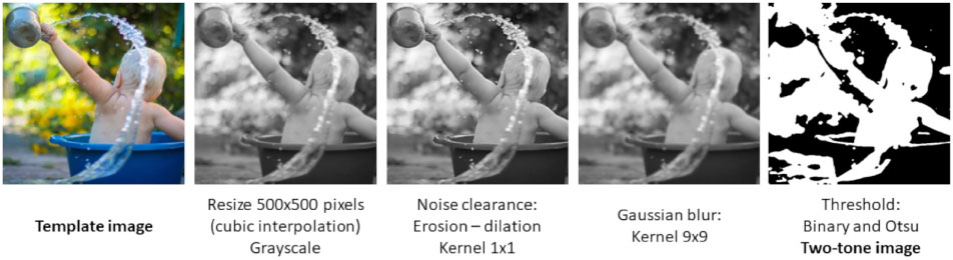
Illustration of the stimulus creation process.

A total of 150 two-tone images were created with a custom Python script from coloured high-definition template images of people and animals downloaded from https://stocksnap.io and https://www.pexels.com/ under the Creative Commons License. Template images were resized to 500 × 500 pixels using cubic interpolation and converted to grayscale. Image noise was cleared through opening (erosion followed by dilation) using a 1 × 1 kernel. A Gaussian blur was then applied with a 9 × 9 kernel. Finally, a binary and Otsu threshold were applied to each image (Sezgin and Sankur, 2004) (Fig. 3).

Resulting two-tone images were piloted with 17 healthy volunteers to assess discrimination sensitivity before and after viewing the templates. Two-tone images that were too easy to disambiguate before viewing the template or too difficult after seeing the template were excluded. From the remaining two-tone images, 60 were selected (30 of people and 30 of animals) for this experiment. The presentation time of 800 ms chosen in our experiment was guided by reaction time data collected during pilot testing. (Seventeen healthy volunteers were asked to indicate the presence of a person in the two-tone stimuli using a keyboard press without any prior information and without presentation time limit. A keyboard press triggered the next stimulus to be presented. Decision time was recorded and 800 ms (mean + 2SD) was chosen for the main experiment.)

### Participant recruitment

We recruited 37 patients with Lewy body disease (LBD: Parkinson’s disease and Dementia with Lewy bodies) from Parkinson’s disease clinics at the National Hospital for Neurology and Neurosurgery, London, UK. All patients satisfied the United Kingdom Parkinson’s Disease Society Brain Bank criteria for Parkinson’s disease, the Movement Disorder Society criteria for Parkinson’s disease dementia or the Dementia with Lewy Bodies Consortium Criteria for Dementia with Lewy Bodies (DLB) (Gibb and Lees, 1988; Emre *et al.*, 2007; McKeith *et al.*, 2017). Patients with LBD were classified as habitual hallucinators (VH) if they scored ≥1 on question two of the Movement Disorder Society Unified Parkinson’s Disease Rating Scale (UPDRS: ‘Over the past week have you seen, heard, smelled or felt things that were not really there?’; Goetz *et al.*, 2008). Seventeen patients with LBD scored ≥1 and were classified as having hallucinations, whilst 20 patients did not.

Further qualitative and quantitative details on the experienced hallucinatory phenomena were collected with the University of Miami Parkinson’s Disease Hallucinations Questionnaire (Papapetropoulos *et al.*, 2008). This quantifies severity (intensity) and frequency of hallucinations. Twenty unaffected controls were recruited from spouses and a volunteer database at our UK centre.

### Motor and psychology assessments

Assessment of motor function was performed using the Unified Parkinson’s Disease Rating Scale (Goetz *et al.*, 2008).

The Mini-Mental State Examination and Montreal Cognitive Assessment were used as measures of general cognition (Dalrymple-Alford *et al.*, 2010; Creavin *et al.*, 2016). To test specific cognitive domains, we used the following assessments (Lezak *et al.*, 2004):

- Attention: Digit span backwards (Wechsler, 2008), Stroop test: Naming subtest (Stroop, 1935)
- Executive function: Stroop Interference (Stroop, 1935), Category fluency (Rende *et al.*, 2002)
- Memory: Word Recognition Task (Warrington, 1984), Logical Memory (immediate and delayed recall) (Wechsler, 2008)
- Language: Graded Naming Task (Warrington, 1997), Letter fluency (Rende *et al.*, 2002)
- Visuospatial: Benton’s Judgment of Line (Benton *et al.*, 1978), Visual Object and Space Perception Battery (Warrington and James, 1991), Hooper Visual Organization Test (Hooper, 1983).

Visual acuity was assessed using the 6-m Snellen chart. All participants had corrected bilateral acuity of ≥6/6. Colour vision was assessed using the D15 (Farnsworth, 1947) and contrast sensitivity using the Pelli-Robson test (Pelli *et al.*, 1988). Smell was assessed using Sniffin’ Sticks (Hummel *et al.*, 1997). The Hospital Anxiety and Depression Scale was used to assess mood (Zigmond and Snaith, 1983) and the REM Sleep Behaviour Disorder to assess sleep (Stiasny-Kolster *et al.*, 2007).

All assessments and the experimental task were performed with patients in the ON state. This avoided potential distress associated with the OFF state, that may have impaired cognitive and perceptual performance and also ensured results were not influenced by between-subject differences in motor performance. Levodopa equivalent daily doses (LEDD) were calculated. The groups were matched for age, gender and time in education. Details of the three study groups are seen in Table 1.

**Table 1:** Study group characteristics

Attribute		Controls *n* = 20	LBD non-VH * n* = 20	LBD VH * n* = 17	*P*-value*
Demographics	Age in years	69.7 (6.9)	68.9 (7.1)	68 (6.9)	0.457^b^
	Male (%)	11 (55)	13 (65)	7 (41.2)	0.079^b^
	Years in education	15.9 (2.6)	15.9 (2.3)	15.4 (2.9)	0.567^a^
Mood (HADS)	Depression score	2.2 (2.2)	**2.5 (2.5)**	**4.5 (2.4)**	**0.017** ^b^
	Anxiety score	4.3 (2.6)	4.5 (4.1)	5.2 (2.9)	0.212^b^
Vision	Visual acuity (bilateral)	1.1 (0.2)	1.1 (0.2)	1.0 (0.2)	0.216^b^
	Contrast sensitivity (Pelli-Robson) (log units) (bilateral)	1.7 (0.2)	**1.7 (0.1)**	**1.5 (0.2)**	**0.017** ^b^
	Colour vision (D15)	0.2 (0.4)	0.8 (1.3)	0.4 (1.3)	0.180^b^
Neuropsychology	MMSE	29.5 (0.7)	29 (1.5)	28 (2.1)	0.057^b^
	MOCA	27.8 (1.2)	26.8 (3.1)	25.5 (3.7)	0.131^b^
Attention	Digit span backwards	8.4 (2.4)	7.2 (2.6)	7.1 (2.2)	0.860^a^
	Stroop: naming (s)	38.1 (6.5)	**35.8 (8.5)**	**44.7 (14.5)**	**0.007** ^b^
Executive function	Stroop: interference (s)	69.1 (14.2)	66.7 (22.9)	83 (27.2)	0.062^b^
	Category fluency	19.7 (4.4)	19.8 (4.3)	17.8 (5.6)	0.219^a^
Memory	Word recognition task	24.8 (0.4)	23.2 (1.9)	23.6 (2.0)	0.219^b^
	Logical memory (delayed)	12.4 (4.3)	10.1 (3.9)	11.1 (2.8)	0.309^a^
Language	Graded naming task	24.4 (2.5)	23.8 (3.9)	22.3 (3.9)	0.079^b^
	Letter fluency	16 (4.7)	14.6 (5.5)	12.8 (4.2)	0.297^a^
Visuospatial	VOSP	56.2 (2.0)	54.6 (3.3)	52.8 (5.4)	0.397^b^
	Benton’s judgement of line orientation	24.4 (4.3)	25.4 (4.1)	22.3 (4.4)	0.071^b^
	Hooper’s visual organization test	25.6 (2.4)	23.2 (3.9)	21.7 (4.9)	0.187^b^
Disease specific	Age at diagnosis		64.4 (9.0)	63.4 (7.3)	0.719^a^
	Disease duration		4.5 (4.6)	4.6 (2.7)	0.944^a^
	RBDSQ		4.2 (2.5)	5.1 (3.0)	0.190^b^
	UPDRS total		**38.7 (13.4)**	**52.8 (15.6)**	**0.003** ^b^
	UPDRS question 1.2			1.76 (0.73)	
	Miami hallucinations questionnaire			5.2 (1.9)	
	UPDRS part 3 (motor score)		24.7 (7.5)	30.5 (10.6)	0.136^b^
	LEDD (mg)		401.9 (288.3)	406 (244.8)	0.500
	Smell test		23.2 (3.9)	21.6 (4.9)	0.082^a^

All data shown (except gender) are mean (SD).

HADS, Hospital anxiety and depression scale; LBD non-VH, patients without visual hallucinations; LBD VH, patients with Lewy body disease and visual hallucinations; LEDD, Total Levodopa equivalent daily dose; MMSE, Mini-mental state examination; MOCA, Montreal cognitive assessment; RBDSQ, REM sleep behaviour disorder screening questionnaire; UPDRS, Unified Parkinson’s disease rating scale; VOSP, Visual Object and Space Perception Battery.

* Uncorrected *P-*values shown are for comparison between LBD/VH and LBD/non-VH (comparison of interest), in bold statistically significant values (*P* < 0.05, uncorrected).

a Student *t*-test.

b Mann-Whitney U test.

The study was approved by the Queen Square ethics committee and all participants provided written informed consent prior to taking part.

### Statistical analysis

We used a two alternative forced choice task where participants were asked to indicate the presence of a person in each presented image. We measured correct responses and reaction times in the Before and After blocks. In addition, we used signal detection theory metrics for analysis. We derived hit rates and false alarm rates from the collected yes/no responses; this was done separately for the Before and After Block. We derived discrimination sensitivity (d′): a measure of the observer’s ability to distinguish ‘signal’ from ‘noise’ distributions (independent of any bias for one or the other), i.e. the separation between them normalized by their variance, and criterion (c): a measure of the bias the observer has to detect a signal (Macmillan and Creelman, 1991). d′ and c were derived from the following equations:

$$\begin{array}{c} d^{\prime }=z\left(h\right)-z\left(f\right)\\ c=-1/2\left[z\left(h\right)+z\left(f\right)\right] \end{array}$$

where h: hit rate, f: false alarm rate.

Independent t-samples and ANOVAs were used to compared normally distributed continuous variables and Mann-Whitney and Kruskall-Wallis tests for non-normally distributed ones. Visual inspection of variable distribution and the Shapiro Wilk test were used to test normality. *Post hoc* correction for multiple comparisons were performed with Tukey test for ANOVA and Nemeyni test for Kruskall-Wallis. In our main comparison of interest (LBD patients with and without hallucinations), we have also performed uncorrected *t*-tests and Kruskall-Wallis tests. This was chosen to avoid missing small yet potentially important differences between the two groups in an effort to minimize Type II error. Statistical significance was set at *P* < 0.05. All statistical analysis was performed in Python 3 using Jupyter Notebook version 5.5.0.

### Data availability statement

The code used to create the two-tone stimuli as well as code used for data analysis is provided in the following repository: https://github.com/AngelikaZa/Increased-weighting-on-prior-knowledge-in-Lewy-Body-associated-visual-hallucinations.-BrainComms2019.git. The specific stimuli used for this experiment are also provided in the same repository. The data that support the findings of this study are available from the corresponding author, upon reasonable request.

## Results

We tested a total of 57 participants; 17 patients with LBD and visual hallucinations (LBD VH), 20 patients with LBD without hallucinations (LBD non-VH) and 20 unaffected, age-matched controls. Three patients with LBD VH had a diagnosis of Dementia with Lewy Bodies and the rest had a diagnosis of Parkinson’s disease, whilst two of the LBD non-VH patients had a diagnosis of Dementia with Lewy Bodies. Apart from diagnostic distinctions, there were no differences between patients with a diagnosis of Dementia with Lewy Bodies and those with Parkinson’s disease in disease duration, clinical or demographic measures. Demographics and clinical characteristics of patients with Dementia with Lewy Bodies and Parkinson’s disease are provided in Supplementary material.

### Prior knowledge leads to greater performance improvement in patients who hallucinate

**Figure 4:**
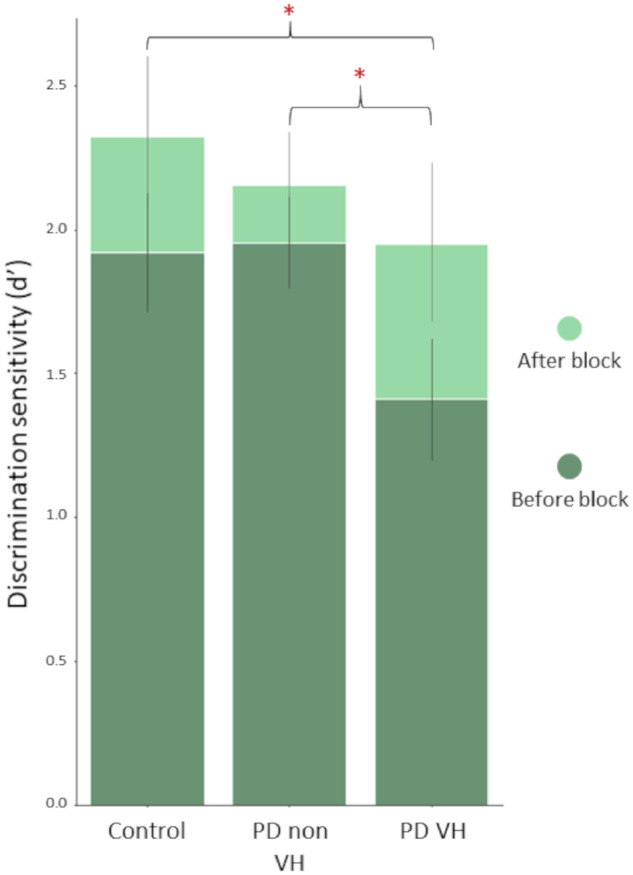
**Improvement in performance in patients with LBD with and without hallucinations and controls.** Discrimination sensitivity (d′) in the Before and After blocks across our three study populations.

As expected by our experimental design, our cohort improved in performance in the After block compared to the Before block both in absolute percentage correct (*t *= 4.12, Hedge’s *g* = 0.77, *P* < 0.001) and discrimination sensitivity, d′ (*t *= 3.73, Hedge’s *g* = 0.69, *P* < 0.001). A between groups ANOVA revealed that this improvement differed between the three study groups in percentage correct: improvement mean ±SD in LBD VH was 6.9% ±4.4 (from 74.5% to 81.5%) compared to 2.7% ±4.4 (from 82% to 84.8%) and 4.7% ±3.5 (from 79.3% to 84%) in controls [*F*(2, 54) = 4.90, *P* = 0.011] and d′: 0.54 ± 0.41 improvement in d′ (from 1.41 to 1.95) in LBD VH compared to 0.2 ± 0.47 (from 1.96 to 2.15) in LBD non-VH and 0.4 ± 0.37 (from 1.92 to 2.32) in controls [*F*(2, 54) = 3.18, *r*^2^ =  0.11, *P* =  0.049]. The improvement in d′ across the three groups is shown in Fig. 4.

**Figure 5:**
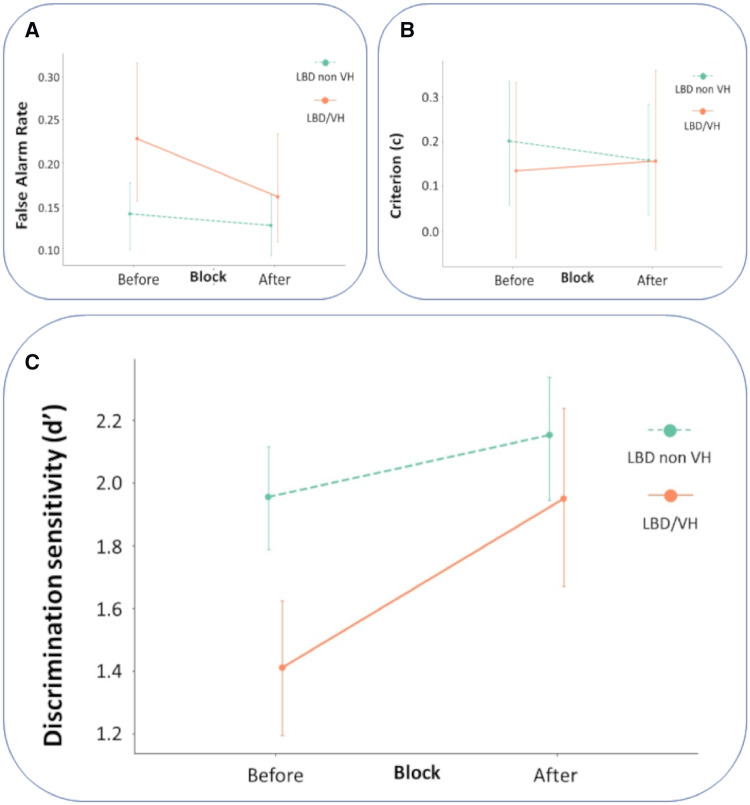
**Improvement in performance in patients with LBD with and without hallucinations.** (**A**) False alarm rates in patients with LBD with and without hallucinations (VH) in the Before and After blocks. LBD/VH showed a significant reduction from Before to After, but LBD non-VH did not (see text). (**B**) Criterion (c) in the Before and After blocks in patients with LBD. There were no group differences in the change from Before to After. Lower c values suggest a response bias to indicate the presence of a person independent of whether a test or control image is shown. (**C**) Discrimination sensitivity (d′) in Before and After blocks in patients with LBD: higher values suggest better participant ability to correctly disambiguate the two-tone images for the presence of a person. LBD/VH show greater improvement in performance from Before to After, compared with LBD non-VH. In all images confidence intervals 95% are shown.

Importantly, planned *post hoc* testing showed that this difference was driven by a difference between patients with LBD with and without visual hallucinations (*t*-test and effect size between LBD non-VH and LBD/VH: *t *= 2.35, Hedge’s *g* = 0.75, *P* = 0.025). Hallucinators improved more than double the amount the non-hallucinators did after viewing the template images: improvement in d′ (d′ diff) mean ±SD = 0.20 ± 0.46 in LBD patients without hallucinations and 0.54 ± 0.41 in those with hallucinations (Fig. 5C). This improvement was seen despite the worse initial performance of hallucinators (d′ in the Before block between LBD non-VH and LBD/VH: *t *= 3.958, Hedge’s *g = *1.27, *P* < 0.001), who in the After block reached the discrimination sensitivity of non-hallucinators (*t *= 1.16, *P* = 0.25). To ensure that the observed difference in improvement was not secondary to a ceiling effect in patients with LBD without hallucinations or controls performing at ceiling in the Before block, we tested the variance of d′ d′ in the Before block between the three groups. Variance was not significantly different in LBD VH compared to LBD non-VH or controls in the Before block (Levene’s test of variance W = 1.09, *P* = 0.344), confirming that neither of the three groups was performing at ceiling in the Before block. The same was true for the After block (W = 0.77, *P* = 0.469). In addition, participants without hallucinations who had better performance in the Before block (higher d′) were still able to improve in the After block; in fact, higher d′ in the Before block was associated with higher improvement in d′ (*r*^2^ = 0.260, df = 40, β = 0.1376, *P* = 0.001). Thus, while ceiling effects in non-hallucinating participants cannot be definitively excluded, the available evidence indicates that such effects are not driving the group differences.

### Performance improvement is driven by a reduction in false alarm rates

The difference in performance improvement was primarily attributed to a greater reduction in false alarm rate in hallucinators: −0.07 ± 0.07 (from 0.23 to 0.16) in LBD VH compared to −0.01 ± 0.08 (0.14 to 0.13) in LBD non-VH (*t *= 2.19, Hedge’s *g = *0.70, *P* = 0.035), Fig. 5A. LBD VH participants had higher false alarm rates in the Before block than LBD non-VH (Mann-Whitney U = 114, *P* = 0.043) but did not differ in false alarm rates in the After block (U = 150, *P* = 0.269). The improvement in hit rate did not significantly differ between LBD with and without VH (*t *= 1.35, *P* = 0.185), nor did the hit rates in the Before (U = 122, *P* = 0.073) or After block (U = 145, *P* = 0.221). Importantly, we did not find any group bias in responses, as criterion was not significantly different across the three groups in either the Before [*F*(2, 54)=0.902, *P* = 0.412] or After blocks [*F*(2, 54)=0.527, *P* = 0.593]. There was also no significant difference in criterion between the Before and After blocks in the whole cohort (*t *= 0.331, *P* = 0.742) or within the three individual groups, reflecting the fact that observers’ received information regarding both test and control stimuli leading to an improved performance in recognizing both the presence and the absence of a person in the After blocks (Fig. 5B).

### Hallucinators and non-hallucinators did not significantly differ in cognitive function

Additionally, all participants underwent extensive neuropsychological, visual and motor assessments to minimize confounders that might influence experiment results. After corrections for multiple comparisons, the three groups did not significantly differ in demographics, time spent in education, low-level vision, general cognitive measures or within specific cognitive domains, including attention, executive functions or language. Of three measures of higher-level visuospatial perception, only one differed between groups: the Hooper test (Kruskal Wallis: *H* = 86, *P* < 0.001) with *post hoc* analysis attributing this difference to lower performance in LBD/VH compared to controls (U = 86, *P* = 0.005) with no difference seen between LBD/VH and LBD non-VH (U = 141, not significant); the other two visuo-perceptual measures were non-significant after correction for multiple comparisons. One of the memory measures differed: Word Recognition task (*H* = 88, *P* < 0.001) but *post hoc* analysis revealed that this was driven by a lower performance in LBD patients compared to controls (U = 100, *P* = 0.001) with no significant difference between LBD/VH and LBD non-VH patients (U = 145, *P* = 0.220). Depression scores were higher in LBD patients with hallucinations (*H* = 24.133, *P* < 0.001) but below the threshold for a diagnosis of depression (Bjelland *et al.*, 2002; Mondolo *et al.*, 2006).

In addition to *post hoc* testing, and to avoid missing out possible group differences, we also performed uncorrected tests in our main comparison of interest: LBD patients with and without hallucinations. The two groups did not differ in demographics, disease duration, age at diagnosis or levodopa equivalent dose. Four patients in our cohort were receiving anticholinesterase inhibitors; two LBD/VH and two LBD non-VH. None were receiving antipsychotic medications. LBD patients with hallucinations had lower contrast sensitivity (U = 106, *P* = 0.017) than those without hallucinations but there were no other statistically significant differences in performance between the two groups in all other visual and visuospatial tasks. Performance in cognitive tasks was also not significantly different, except for one measure of executive function (Stroop: Naming; U = 89, *P* = 0.007). Finally, LBD patients with and without hallucinations differed in total Unified Parkinson’s Disease Rating Scale (which measures motor and non-motor symptoms; U = 80.5, *P* = 0.003), with patients with hallucinations having higher scores compared to patients without hallucinations signifying higher levels of disability. However, the objective motor component of the Unified Parkinson’s Disease Rating Scale did not differ between the two groups (U = 1.867, *P* = 0.14). The demographics and results of clinical assessments in our cohort are seen in Table 1.

### Performance improvement increases with hallucination severity

To better quantify the effect of prior knowledge in patients with LBD who hallucinate, we also collected qualitative and quantitative data on hallucination attributes and severity. Of the 17 patients with LBD and VH, 4 (23.5%) had provoked, 3 (17.3%) formed, and 10 (58.8%) animate hallucinations; on a validated quantitative scale of hallucination severity (Papapetropoulos *et al.*, 2008) the mean ±SD score was 5.2 ± 1.9. Importantly, in people with LBD and hallucinations performance improvement was correlated with hallucination severity (*r*^2^ = 0.617, df = 16, *P* < 0.001). The correlation remained significant after correcting for the observed differences in contrast sensitivity, Stroop naming scores, depression scale scores and Unified Parkinson’s Disease Rating Scale as well as Mini-Mental State Examination, levodopa equivalent dose and Hooper (visuospatial measure) (df = 7, *t *= 2.549, *P* = 0.038).

Given that our group of participants included a subgroup with dementia with Lewy bodies, we performed an additional analysis, with those five patients excluded, to test whether effects were driven solely by that subgroup. Even with this subgroup of patients with DLB excluded, we found that d′ was significantly higher in hallucinators compared to non-hallucinators (improvement in d′: 0.62 ± 0.41 in Parkinson’s disease/VH compared to 0.27 ± 0.43 in Parkinson’s disease non-VH, *t *= 2.35, *P* = 0.025). This was still due to a higher reduction in false alarm rates in hallucinators than non-hallucinators (*t* = −2.33, *P* = 0.027) rather than a higher increase in hit rates (*t *= 0.95, *P* = 0.352). Finally, hallucination severity was still associated with higher improvement in d′ in the group of Parkinson’s disease hallucinators: *r*^2^ = 0.733, df = 13, *t *= 6.282, *P* < 0.001.

## Discussion

Our study shows that patients with LBD VH place relatively higher weighting on prior knowledge in perceptual inference. Improvement in our visual disambiguation task was more than double in patients with LBD who hallucinate compare to those who do not, with hallucinators reaching the same discrimination sensitivity as non-hallucinators and controls despite worse initial performance.

Our findings provide mechanistic insights into LBD-associated hallucinations. Hallucinations in LBD are usually progressive, starting as minor illusions or misperceptions before complex, detailed or animate hallucinations emerge (Mosimann *et al.*, 2004; Weil *et al.*, 2016). Whilst initially patients have full insight into their symptoms, insight is often lost with disease progression and delusional ideas around hallucinations can develop (Weil *et al.*, 2016). We found that severity of hallucinations was associated with greater improvement in performance and therefore with greater effects of prior knowledge. This may also provide some insights into how hallucinations may progress during the disease. Changes in the whole visual system, from the retina to the visual cortex, are seen in LBD with some evidence suggesting that, at least some, changes occur early in the disease course (Williams-Gray *et al.*, 2009; Erskine *et al.*, 2019). A possible explanation for hallucinations is that early damage in the low-level visual system could result in a loss of signal/noise, i.e. decreased sensory precision. Decreased sensory precision is consistent with drift diffusion modelling findings of a decreased drift rate (slower evidence accumulation) in LBD/VH relative to LBD (O’Callaghan *et al.*, 2017*a*). That study did not directly assess the effects of visual priors but here, we have shown an increased relative weighting of prior knowledge. This was not due to bias (lower criterion) towards perceiving people in LBD/VH. Prior knowledge could be afforded higher weighting in LBD/VH to compensate for reduced sensory precision. We saw higher false alarms and lower d′ in LBD/VH patients at baseline, implying the ‘signal’ and ‘noise’ distributions are closer in LBD/VH but we found no evidence of bias (lower criterion) towards perceiving people in LBD/VH. Rather, we saw an increase in false alarms and lower d′, implying the ‘signal’ and ‘noise’ distributions are closer in LBD/VH. Strikingly, the benefit of viewing the colour pictures in LBD/VH was mostly realized as a reduction in false alarms rather than an increase in hits.

The observed reduction in false alarm rate in the After blocks with the same hit rate and criterion could be explained by an increase in signal strength combined with a change in the absolute cut-off LBD/VH participants use to make their decision (criterion shift). In an equal variance signal detection theory model, a simpler explanation is that prior knowledge leads to sensory sharpening or lowering of the noise distribution which has been previously suggested in a similar task (Teufel *et al.*, 2018): this effect may be greater in those with least sensory precision. In addition, it is still unclear whether hallucinations in LBD/VH are uniquely ascribable to a loss of sensory precision is unclear: it could also be that the precision of these patients’ prior beliefs increases in absolute terms (alongside any sensory precision loss). We did not see strong evidence for sensory precision loss in the low-level visual tasks in LBD/VH, but to assess the relative contributions of priors and likelihoods one needs a task designed to measure both separately (Karvelis *et al.*, 2018).

Importantly, we have shown that the striking performance improvement seen in LBD patients with hallucinations cannot be fully attributed to a ceiling effect in non-hallucinating groups. Hallucinators did not significantly differ from non-hallucinating groups in variance of discrimination sensitivity. A lower variance in the LBD non-VH and controls would be expected if the main driver in improvement was a ceiling effect across all groups. More importantly, individually good performance in the Before block was associated with higher improvement in discrimination sensitivity; should a ceiling effect be present we would expect the highest performers in the Before block being unable to improve as much in the After block. Despite this, it is still possible that the observed improvement is driven by worse disambiguation in the Before block in hallucinators compared to non-hallucinating groups, rather than a stronger effect of prior knowledge. However, within the group of hallucinators, worse hallucination severity was associated with higher improvement in d′; this could not be explained by group differences in disambiguation.

Interestingly, we observed wide variability in performance improvement in patients who did not experience hallucinations. In addition, performance improvement was significantly correlated with hallucination severity. This raises the question of whether patients with Parkinson’s disease with greater improvement in performance are on the cusp of developing hallucinations in the near future. Prospective studies of sensory accumulation and prior knowledge in people with LBD could shed further light on this question and evaluate the ability of tests of prior knowledge such as ours as potential markers of susceptibility to hallucinations.

Our LBD subjects were quite evenly matched in terms of their vision and cognitive performance. Low-level vision is affected in Parkinson’s disease with reduce visual acuity, contrast sensitivity and colour vision reported, whilst both pathological and optical coherence tomography studies confirm changes in the retina, the earliest part of the visual system (Hajee *et al.*, 2009; Lee *et al.*, 2014; Weil *et al.*, 2016). Higher order visual processing is also affected in LBD (Mori *et al.*, 2000; Mosimann *et al.*, 2004; Weil *et al.*, 2016). Both low- and high-level visual processing is more affected in patients with LBD who experience hallucinations (Diederich *et al.*, 2005) although hallucinations can occur even in patients with equivalent visual performance (Gallagher *et al.*, 2011). Hallucinations in LBD are also linked with worse cognitive impairment (Fénelon *et al.*, 2000; Weil *et al.*, 2016). However, in our cohort, cognition in LBD VH was not significantly worse than in LBD without hallucinations across all cognitive domains, with worse performance in LBD/VH in only one measure of executive function. In addition, LBD/VH were poorer than LBD patients without hallucinations in one low-level visual task (contrast sensitivity) but we found no difference between LBD/VH and LBD without hallucinations in other tests of visual function.

We studied patients with Parkinson’s disease and Dementia with Lewy Bodies together due to their indistinguishable end-phenotype and common pathological features; such an approach has been advocated in recent years (Jellinger, 2012; Postuma *et al.*, 2016; Weil *et al.*, 2017). Considering the two diseases together can provide useful insights into common mechanisms leading to the same symptom of hallucinations. In our cohort, within the group of VH and the group of non-VH participants, patients with an underlying diagnosis of Dementia with Lewy Bodies and those with a diagnosis of Parkinson’s disease did not significantly differ in any clinical or cognitive measure (beyond diagnostic characteristics) and we continued to find the same effects when we examined our group without the DLB patients. However, underlying pathological differences could potentially have different effects on top-down and bottom-up pathways in the two conditions. Further studies in a more homogenous pathological population might elucidate these.

We performed all assessments and the experimental task with patients in the ON state, with patients taking their usual medications, to prevent potential confounds arising from motor differences and the distress of being OFF. Although levodopa equivalent doses did not differ between patients with and without hallucinations, further studies could specifically test LBD patients ON and OFF medication to examine the effect of dopamine on sensory integration. This is particularly relevant given the potential higher risk of VH with high levodopa doses in LBD (Fénelon *et al.*, 2000; Gallagher *et al.*, 2011; Weil *et al.*, 2016) and the link between striatal dopamine release and hallucinations (Cassidy *et al.*, 2018). Indeed, in Parkinson’s disease (without hallucinations), levodopa has been shown to increase the weighting of sensory evidence in a visual decision-making task (Vilares and Kording, 2017). This apparent contradiction—of dopamine appearing to promote hallucinations (top-down) and sensory evidence (bottom-up)—might be resolved if one considers that the benefit of increasing sensory weighting depends on the quality of the data. If this weighting benefits precise sensory data, then sensory input will contribute more to inference (Vilares and Kording, 2017), but if it merely amplifies sensory noise, then hallucinations may increase.

Finally, our findings are consistent with studies in psychiatric illness and hallucination-prone individuals. People at risk of or with early psychosis also exhibit a shift towards prior knowledge and have a perceptual advantage in disambiguating a degraded visual scene or degraded speech (Teufel *et al.*, 2015; Alderson-Day *et al.*, 2017; Davies *et al.*, 2018). Our study in combination with these findings suggests that hallucinations may share a computational mechanism across diseases with the same neural systems involved in generating hallucinations regardless of the pathophysiological diagnosis. This could have useful implications in translating advances and treatments across fields.

In summary, we show that VH in LBD are associated with an increased use of prior knowledge when viewing ambiguous visual stimuli, with increased hallucination severity predicting greater effects of prior knowledge. The shift from sensory evidence to prior knowledge in visual perception may be a useful marker for hallucination severity in LBD as well as other neurodegenerative and psychiatric illnesses where hallucinations are prominent.

## Supplementary Material

fcz007_Supplementary_Data

## Abbreviations

HADS: Hospital Anxiety and Depression Scale
LBD: Lewy body disease
LEDD: Levodopa equivalent daily dose
MMSE: Mini-Mental State Examination
MOCA: Montreal Cognitive Assessment
RBDSQ: REM Sleep Behaviour Disorder
UM-PDHQ: University of Miami Parkinson's Disease Hallucinations Questionnaire
UPDRS: Unified Parkinson’s Disease Rating Scale
VH: Visual hallucinations
