# Supplementary material for: Increased weighting on prior knowledge in Lewy body-associated visual hallucinations
Source: Brain Commun. 2019 Jul 16;1(1):fcz007. doi: 10.1093/braincomms/fcz007 (PMC6924538; doi:10.1093/braincomms/fcz007)
Supplement: fcz007_Supplementary_Data [file fcz007_supplementary_data.docx]

**Supplementary Material**

**Table 1. Group characteristics, comparison between participants with Parkinson’s disease and patients with Dementia with Lewy bodies**

| **Attribute** | | **PD**  **n = 32** | **DLB**  **n = 5** | **p value*** |
| --- | --- | --- | --- | --- |
| **Demographics** | Age in years | 69.3 (7.2) | 67.8 (6.4) | 0.817^a^ |
|  | Male (%) | 17 (40.1) | 3 (60) | 0.398^b^ |
|  | Years in Education | 15.6 (2.6) | 16.0 (3.2) | 0.730^a^ |
| **Mood (HADS)** | Depression score | **3.2 (2.5)** | **5.3 (3.3)** | **0.033**^b^ |
|  | Anxiety score | **4.4 (3.7)** | **5.8 (2.1)** | **0.043^b^** |
| **Vision** | Visual acuity (bilateral) | 1.1 (0.2) | 1.0 (0.2) | 0.339^b^ |
|  | Contrast sensitivity (Pelli Robson) (log units) (bilateral) | 1.6 (0.2) | 1.5 (0.2) | 0.168^b^ |
|  | Colour vision (D15) | 0.5 (1.2) | 1.5 (1.9) | 0.052^b^ |
| **Neuropsychology** | MMSE | **28.9 (1.5)** | **26.4 (2.3)** | **0.005^b^** |
|  | MOCA | **27.1 (2.4)** | **20.6 (3.8)** | **0.002^b^** |
| Attention | Digit span backwards | 7.3 (2.3) | 6.0 (2.9) | 0.257^a^ |
|  | Stroop: Naming (sec) | **25.2 (7.9)** | **39.4 (12.1)** | **0.029^b^** |
| Executive function | Stroop: Interference (sec) | **69.6 (21.4)** | **118.8 (28.2)** | **0.002^b^** |
|  | Category fluency | **19.8 (4.5)** | **13.0 (3.9)** | **0.003^a^** |
| Memory | Word Recognition Task | **23.7 (1.6)** | **21.4 (3.0)** | **0.036^b^** |
|  | Logical Memory (delayed) | **11.6 (2.7)** | **6.8 (1.5)** | **0.001^a^** |
| Language | Graded Naming Task | **23.5 (3.7)** | **19.8 (4.1)** | **0.026^b^** |
|  | Letter Fluency | **14.6 (4.6)** | **14 (3.6)** | **0.007^a^** |
| Visuospatial | VOSP | 54.0 (4.3) | 52.0 (4.9) | 0.420^b^ |
|  | Benton’s Judgement of Line Orientation | 23.8 (3.9) | 16.7 (2.3) | 0.328^b^ |
|  | Hooper’s Visual organisation test | 22.9 (4.3) | 19.9 (4.6) | 0.073^b^ |
| **Disease specific** | Hallucinations (%) | 14 (33.3) | 3 (60.0) | 0.480^b^ |
|  | Miami Hallucinations Questionnaire | 2.2 (2.8) | 3.8 (3.8) | 0.154 |
|  | Disease duration | 4.8 (3.8) | 2.4 (2.3) | 0.168^a^ |
|  | RBDSQ | 4.3 (2.7) | 6.2 (3.0) | 0.073^b^ |
|  | UPDRS Total | 44.8 (16.0) | 47.2 (16.9) | 0.395^b^ |
|  | UPDRS part 3 (motor score) | 27.4 (9.9) | 28.4 (7.1) | 0.420^b^ |
|  | LEDD (mg) | 409.1 (257.8) | 370.0 (343.4) | 0.764^a^ |
|  | Smell test | 7.5 (3.2) | 6.0 (3.0) | 0.258^a^ |
| *PD: Parkinson’s Disease; DLB: Dementia with Lewy Bodies; VH: Visual hallucinations.*  *All data shown (except gender) are mean (SD).*  ^a^ Student t test. ^b^Mann-Whitney test.  *HADS: Hospital anxiety and depression scale; MMSE: Mini-mental state examination; MOCA: Montreal cognitive assessment; VOSP: Visual Object and Space Perception Battery UPDRS: Unified Parkinson’s disease rating scale; LEDD: Total Levodopa equivalent daily dose; RBDSQ: REM sleep behaviour disorder screening questionnaire.* | | | | |

Importantly within the subgroup of participants with hallucinations, patients with Parkinson’s disease and patients with Dementia with Lewy Bodies did not differ in any of the above measures except for lower MOCA in patients with DLB (U=7.0, p=0.043), worse Stroop interference time in DLB (U=7.0, p=0.044) and worse performance on Logical Memory Test (U=1.0, p=0.006).
